# Evaluation of vitamin D supplementation intake among children; cross-sectional observational study

**DOI:** 10.12688/f1000research.123373.1

**Published:** 2022-12-08

**Authors:** Niloufar Sharafi, Aiman Fatima, Syed Wasif Gillani, Nour Kaddour, Rawa Banoori, Riham Mohamed Elshafie, Hassaan Anwer Rathore

**Affiliations:** 1 College of Pharmacy,, Gulf Medical University,, Ajman, United Arab Emirates; 2 Clinical and Hospital Pharmacy Department, College of Pharmacy, Taibah University, Al Madinah Al Munawwarah, Saudi Arabia; 3 Clinical Pharmacy Department, ASUSH,, Ain Shams University, Cairo, Egypt; 4 College of Pharmacy, QU Health, ,, Qatar University, Doha,, 2713,, Qatar

**Keywords:** Vitamin D, deficiency, frequency, supplementation, dietary sources, sunlight exposure, challenges

## Abstract

Background: The purpose of this study was to review the vitamin D supplementation intake status among children in the general public, determine the vitamin D supplements practices, and the barriers that parents and children face with supplementation.

Methods: A cross-sectional observational questionnaire-based survey study design was used. A convenience sampling technique was used to collect the data. An online Rao soft sample size calculator was applied to determine the sample size of 319. The response rate of participants was expected to be 63%, the margin of error was 5% and the level of confidence was 95%.

Results: A total of 248 parents (89.1% mothers (n =203)) and 15.7% fathers (n=39) with a mean ± SD age of 35.4 ± 7.04 years, completed the study (77.7% response rate). Parents reported that the supplements used the most by children were vitamin D supplements (21.85%) and multivitamins (21.8%) followed by calcium supplements (5.6%). However, 27.8% of children in this study did not take any supplements. Of all the parents, 65% (162) of them reported sending their child outside to play while 34.67% (86) of parents had reported no outdoor activity. Approximately 184 (74.2%) parents reported the child’s diet to contain multiple natural sources of vitamin D. However, 69 (27.8%) parents reported giving none of the natural sources of vitamin D to their children through the diet. Parents with higher education about 62.9% (n=156) had a higher frequency of providing vitamin D supplements to their children. Children in high-income families (43.63%) were more likely to take vitamin D supplements than those in middle- or low-income families.

Conclusion: The study concluded that challenges like the educational and financial background of parents, family-income level, and health insurance status could help aid in addressing the overall burden of vitamin D deficiency among young children.

## Introduction

Vitamin D is known among the critical minerals to play an important role in maintaining normal body functions. ^1^ It allows bone mineralization and avoids hypocalcemic tetany (such as involuntary muscle contraction, cramps, spasms, etc). ^2^ It is also known for aiding osteoblasts and osteoclasts in developing and remodeling the bone preventing it from being brittle. ^3^ Other functions of vitamin D in the body include inflammation reduction and regulation of cell growth, neuromuscular and immune function, and glucose metabolism. ^4^ Vitamin D also affects the expression of several genes that code for proteins that govern cell proliferation, differentiation, and apoptosis. Vitamin D receptors can be found in many tissues, and some of them transform 25(OH) D to 1,25 (OH) D. ^5^

Maintaining optimum levels of calcium and vitamin D during childhood and adolescence is critical for bone growth. ^6^ Vitamin D is said to lower the risk of cancer, prevent viral infections, alleviate musculoskeletal pain, and calm mood disorders including depression, according to some claims. There has also been a surge in scientific interest in studying vitamin D at both the basic and clinical levels to address these and other claims. ^7^ Children with vitamin D deficiency develop a disease known as rickets, which is characterized by a frame and fragile bone, making the legs appear bent. ^8^ Vitamin D has been shown to reduce the risk of premature birth in pregnant women. ^9^

A child's vitamin D deficiency can start as early as birth, which can damage not just their bone metabolism but also their immunological system, making them more susceptible to illnesses early in life. ^10^ For the treatment of vitamin D deficiency rickets, the American Academy of Pediatrics (AAP) recommends an initial two- to three-month regimen of “high-dose” vitamin D therapy of 1000 units daily in neonates, 1000 to 5000 units daily in infants one to 12 months old, and 5000 units daily in patients over 12 months old. ^11^

Epidemiologic studies, at least in adults, apart from the risk of osteomalacia and osteoporosis, have associated hypovitaminosis D with an increased risk of several cancers, autoimmune diseases (type 1 diabetes, multiple sclerosis, rheumatoid arthritis, and Crohn’s disease), heart disease, hypertension, metabolic syndrome, asthma, upper respiratory tract infections, muscle weakness, and falling. ^12^ The pleiotropic action of vitamin D was already revealed on molecular, cellular, tissue, and organ levels. ^13^ These observations modified the current knowledge about vitamin D metabolism and methods of diagnosis of vitamin D deficiency states. ^14^

Unfortunately, vitamin D is found rare in food. ^15^ Vitamin D is found in only a few foods. Fish liver oils and the meat of fatty fish (such as trout, salmon, tuna, and mackerel) are among the greatest sources. The amount of vitamin D in a human's tissue is influenced by its food. Vitamin D is also found in modest levels in beef liver, egg yolks, and cheese, mostly in the form of vitamin D3 and its metabolite 25(OH)D3. ^16^ Vitamin D2 is found in varying levels in mushrooms. Some commercially available mushrooms have been exposed to UV radiation to boost their vitamin D2 levels. In addition, the FDA has approved UV-treated mushroom powder as a food additive for use as a vitamin D2 source in food items. ^17^

Furthermore, vitamin D is added to milk, many ready-to-eat bowls of cereal, and some yogurt and orange juice brands. It is found in modest concentrations in cheese and some margarine. ^18^

In the U.A.E., the consistent predominant hot weather, inadequate exposure to sunlight, and low nutritional intake of vitamin D result in low serum concentrations of circulating 25(OH) D, a condition known as hypovitaminosis D. ^19^ Furthermore, recent lifestyles involving using cars for transport over walking, and children indulging in electronics and staying indoors have also influenced low vitamin levels. Low dietary intake of vitamin D and calcium, and other factors, including obesity and low social status, are all associated with low serum levels of vitamin D. ^20^^–^^22^

Further research is needed to be conducted on the production of high-potency–food-based vitamin D supplements, the move to mandatory fortification of cereal grain staples, and the development of natural food sources with higher vitamin D content are all potentially safe and efficient pathways for overcoming the barriers to optimal vitamin D status. Although various studies suggest a high prevalence of vitamin D deficiency among adults and children, no randomized controlled trials have been fully performed on vitamin D deficiency and supplementation among children in the UAE. When compared to the expense of providing therapies for many chronic diseases closely linked to vitamin D deficiency, taking vitamin D supplements is a much better alternative.

The purpose of this study was to review the vitamin D supplementation intake status among children in the general public, to evaluate the vitamin D supplements practices, the natural sources of vitamin D from their diet, and the barriers that parents and children face with supplementation.

## Methods

### Study design and setting

A cross-sectional observational questionnaire-based survey study design was used in this study. The survey was conducted in public places in the U.A.E. The data was collected over seven months from October 2021 to April 2022.

### Research tool

The questionnaire was adapted from the literature and evaluated for content validation.

Part one of the questionnaire consists of demographic information (e.g. age, nationality, gender, etc.)

The second part collected information about supplementation intake and natural sources of vitamin D intake (e.g, if the child has milk, yogurt, etc. in his diet).

The last part consists of the outdoor activity level of the child (e.g. hours the child spends playing outdoors).

All the parts of the data were collected based on yes or no or multiple response questions. ^23^

### Variables

Vitamin D deficiency, education level of parents, outdoor activity hours of children, health insurance status of parents, income level of the families.

### Primary outcome

• The primary outcome of this study was to observe the vitamin D supplementation intake status.
• The possible reasons for vitamin D deficiency among children.
  Parents may have difficulties in supplementing their children's diets due to their financial level, health insurance status, and level of literacy.

### Participants and sampling method

A convenient sampling technique was used to collect data from approximately 248 participants from public places in Ajman, U.A.E. An online Rao soft sample size calculator was applied to determine the sample size, which was 319. The response rate of parents was expected to be 63%, the margin of error was 5% and the level of confidence was 95%. ^24^ Around 319 participants were expected and 248 responded and actively participated in the study, 77.7% was the response rate of the study participants.

### Missing data

This study has no missing data.

### Inclusion criteria

Participants who had at least one child between 4-15 years of age and who agreed to participate.

### Exclusion criteria

Children with minor illnesses that are common in the general population and those suspected clinically of having rickets. Children with cognitive and behavioral disorders were excluded from the study.

### Ethical issues

Ethics approvals have been obtained for the study. This is the ethics approval number IRB/COP/STD/74/Oct-2021 from GMU.

### Consent form

The questionnaire content was described before giving it to the parents and the written consent form was taken from each participant. The consent form was as follows:

“Your participation in this survey is voluntary. You may choose not to participate. If you decide to participate in this survey, you may withdraw at any time. If you decide not to participate in this study, or if you withdraw from participating at any time you will not be penalized. Filling out this form means that you accept to participate in this research.”

### Statistical analysis

The data analysis was done using the SPSS statistical package for social sciences software. A Chi-square analysis was done. Mean, standard deviation, and mean comparison was utilized for continuous data. Both a tabular and graphic version of the data was used to show it. A 5% degree of confidence and a 0.5 margin of error were chosen.

### Bias

There is no bias in any trend in the collection, analysis, interpretation, or review of the data that can lead to conclusions that are different from the truth.

## Results

The study reported no missing data. A total of 248 parents (89.1% mothers (n=203)) and 15.7% fathers (n=39) with a mean±SD age of 35.4±7.042 years, completed the study. About 62.9% (n=156) parent were holding a university degree, while 26.2% (n=65) of the participants completed a secondary school, 9.7% (n=24) completed primary school and 1.2% (n=3) of parent were uneducated. Moreover, almost 61.7% (n=153) of mothers were unemployed, while a smaller number of mothers 27% (n=67), and 11.3% (n=28) were employed and employed with medical background. For fathers the 70.2% (n=174) were employed and 21% (n=52), 4.8% (n=12) and 4% (n=10) were self-employed, employed with a medical background and unemployed, respectively. Approximately 42.7% (n=106) participants had income level of more than 10,000, while 35.5% (n=88), 10.1 (n=25) and 4.8 (n=12) had income level of 5,000-10,000, 2,000-5,000 and less than 2,000, respectively. More than half of the participants has insurance and only a few participants 28.6% (n=71) had no insurance ( Table 1).

**Figure:**
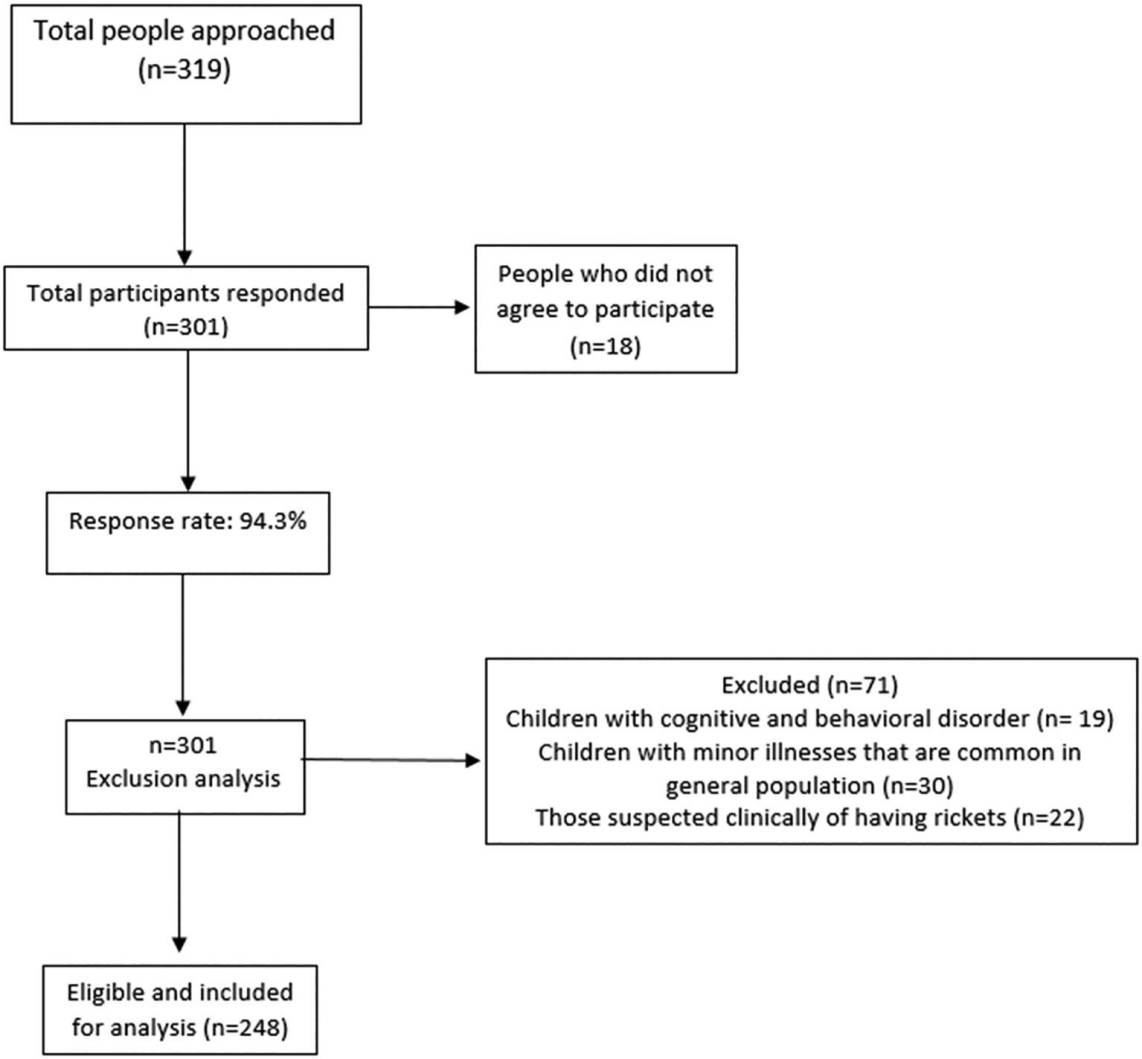
Study flow diagram.

**Table 1.** Sociodemographic parameters of the study participants.

Characteristics	N(%)
Gender	
Mother	203(81.9)
Father	39(15.7)
Others *	6(2.4)
Age (mean±S.D.)	35.4±7.042
Education Level	
Not educated	3(1.2)
Primary School	24(9.7)
Secondary School	65(26.2)
University	156(62.9)
Father Employment	
Employed	174(70.2)
Unemployed	10(4.0)
Self-employed	52(21)
Employed with Medical Background	12(4.8)
Mother Employment	
Employed	67(27)
Unemployed	153(61.7)
Employed with Medical Background	28(11.3)
Income	
Level Less than 2,000	12(4.8)
2,000-5,000	25(10.1)
5,000-10,000	88(35.5)
More than 10,000	106(42.7)
Insurance	
Government	51(20.6)
Private	126(50.8)
None	71(28.6)

* Caregivers.

**Figure 1.**
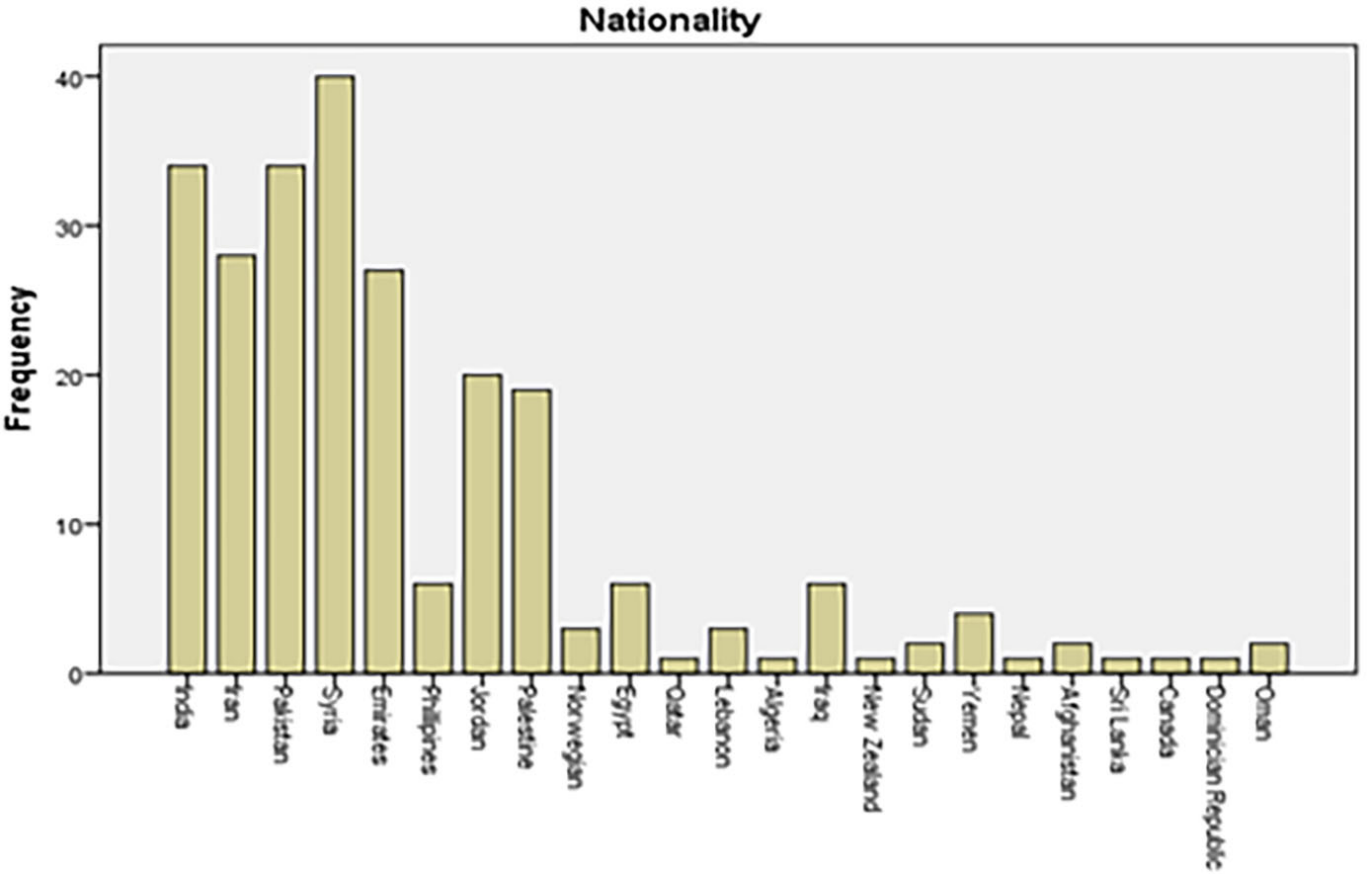
Nationality distribution of study participants.

This research has participants from different countries (total=23). The majority (67%) of participants are from five countries (India, Iran, Pakistan, Syria, and Emirates). The completed data has been presented in Figure 1.

A higher proportion of children received supplements whose parents were educated to the level of secondary school and above. Table 2 shows the literacy level of parents and supplementation.

**Table 2.** Literacy level of participants and supplementation practice.

		Supplements taken			
		Vitamin D	Calcium supplements	Multivitamins	Multiple sources
		N(%)	N(%)	N(%)	N(%)
Education level	Not educated	1(1.85)	1(7.14)	1(1.85)	0
	Primary school	5(9.25)	0	11(20.37)	3(5.35)
	Secondary school	15(27.77)	9(64.28)	10(18.51)	18(32.14)
	University	33(61.11)	4(28.57)	32(59.25)	35(62.5)

The data on outdoor activity levels included the average frequency of outdoor activity per day. Of all the parents, 65% (162) of them reported sending their child outside to play while 34.67% (86) had no outdoor activity. The mean hours of outdoor activity for the children were 2.046 ±1.61. It was found that on average, children spent 0.15-6 hours playing outside in the sun therefore exposed to sunlight ( Table 3).

**Table 3.** Outdoor activity level of children.

Characteristics	N(%)
Activity	
Yes	162(65)
No	86(34.67)
Hours mean±S.D.	2.046±1.61
Min- Max (hours)	0.15-6 hours

**Figure 2.**
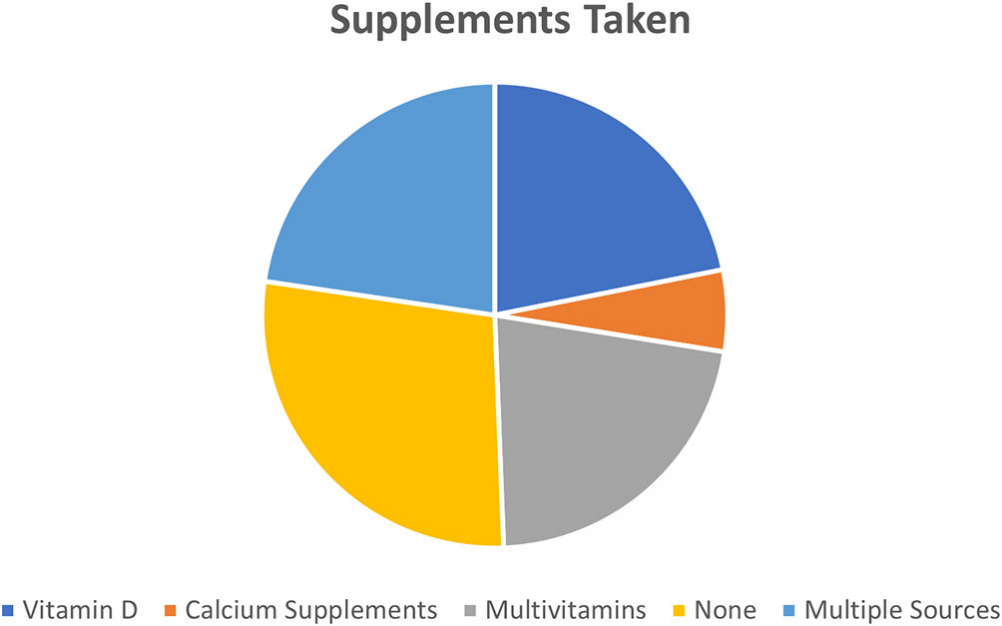
Supplementation intake among the study participants.

Parents reported that supplements used the most by children were Vitamin D supplements (21.85%) and multivitamins (21.8%) followed by calcium supplements (5.6%). However, 27.8% of children in this study did not take any supplements. While other parents reported mixed intake of supplements (for example some children took vitamin D plus calcium supplements while others took vitamin D and multivitamins ( Figure 2).

**Figure 3.**
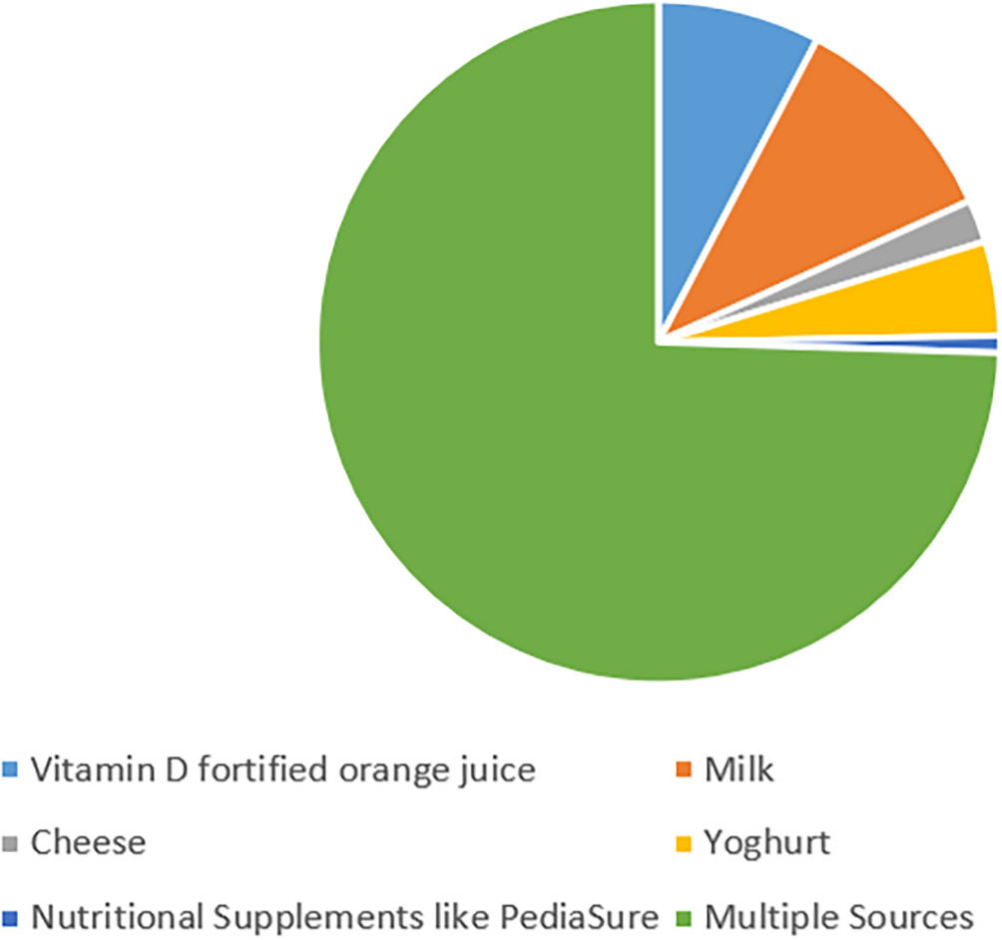
Children’s intake of natural sources containing vitamin D.

Figure 3 summarizes and describes that out of the 248 participants, 184 (74.2%) parents reported their child’s diet to contain multiple natural sources of vitamin D (for example some children had milk plus cheese in their diet while others had yogurt plus cheese plus vitamin D fortified orange juice). However, 69 (27.8%) parents reported giving none of the natural sources of vitamin D to their children through the diet.

Children in high-income families (43.63%) were more likely to receive vitamin D supplements than those in middle- or low-income families ( Table 4).

**Table 4.** Participant’s income level and supplementation practices.

		Supplements taken				
		Vitamin D	Calcium supplements	Multivitamins	Multiple sources	None
		N(%)	N(%)	N(%)	N(%)	N(%)
Income level	Less than 2,000	5(9.09)	0	6(11.11)	1(1.78)	2(2.89)
	2,000-5,000	8(14.54)	0	8(14.81)	6(10.71)	8(11.59)
	5,000-10,000	18(32.72)	7(50)	17(31.48)	21(37.5)	30(43.47)
	More than 10,000	24(43.63)	7(50)	23(42.59)	28(50)	29(42.02)

Parents with private health insurance 51.85% were more likely to give vitamin D supplements to their children compared to those with government health insurance 25.92% and no health care insurance 22.22% ( Table 5).

**Table 5.** Health insurance status of study participants and vitamin D supplementation intake.

		Supplements taken			
		Vitamin D	Calcium supplements	Multivitamins	Multiple sources
		N(%)	N(%)	N(%)	N(%)
Insurance	Governmental	14(25.92)	0	14(25.92)	5(8.92)
	Private	28(51.85)	14(100)	20(37.03)	38(67.85)
	No insurance	12(22.22)	0	20(37.03)	13(23.21)

The study included participants from 23 various nationalities. The top five countries with the most participants were, India, Pakistan, Syria, and the U.A.E. Out of 34 participants from India, only 11 (32.35%) reported the use of vitamin D supplementation by their children, four (11.76%) reported using calcium supplements, six (17.64%) reported using multivitamins and the other 6 of them (17.64%) reported giving multiple sources of vitamins to their children.

However, out of the 34 participants from India, seven (20.58%) did not use any vitamin supplements at all. While out of 28 participants from Iran, only 6 participants, (21.41%) reported the use of vitamin D supplementation by their children, three (10.71%) reported using calcium supplements, ten (35.71%) reported using multivitamins and the other eight of them (28.57%) reported giving multi-sources of vitamins to their children. Among the 34 participants from Pakistan, the lowest amount of vitamin D supplements intake, only four (11.76%) participants reported giving supplements to their children and three (8.82%) reported using calcium supplements, only one (2.94%) parent reported using multivitamins and the other 12 of them (34.29%) reported giving multi-sources of vitamins to their children. Out of 27 participants from Emirates, few participants which are five (18.51%) of them reported the use of vitamin D supplementation by their children, two (7.4%) reported using calcium supplements, seven (25.94%) reported using multivitamins and the rest 11 (40.74%) reported giving multi-sources of vitamins to their children. The participants from Syria reported the highest vitamin D intake among the countries, out of 40 participants from India, only 12 (30.76%) reported the use of vitamin D supplementation by their children, one (2.56%) reported using calcium supplements, nine (23.07%) reported using multivitamins and the other 10 (25.64%) reported giving multiple sources of vitamins to their children Table 6.

**Table 6.** Nationality of study participants [top 5=163 (67%)] and vitamin supplements.

	Supplements taken				
	Vitamin D	Calcium supplements	Multivitamins	Multiple sources	None
	N(%)	N(%)	N(%)	N(%)	N(%)
India (N=34)	11(32.35)	4(11.76)	6(17.64)	6(17.64)	7(20.58)
Iran (N=28)	6(21.42)	3(10.71)	10(35.71)	1(3.57)	8(28.57)
Pakistan (N=34)	4(11.76)	3(8.82)	1(2.94)	12(34.29)	14(41.17)
Syria (N=40)	12(30.76)	1(2.56)	9(23.07)	10(25.64)	7(17.94)
Emirates (N=27)	5(18.51)	2(7.4)	7(25.92)	11(40.74)	2(7.4)

## Discussion

This cross-sectional study demonstrates that inadequacy of vitamin D remains a risk in the U.A.E due to its geographical location being at the equator leading to the hot harsh climate throughout the year limiting the children’s exposure to sunlight. Depending on the effectiveness of UVB photons to promote vitamin D production, the amount of sun exposure necessary for the creation of ultraviolet B-induced vitamin D in the skin impacts cutaneous synthesis. ^25^ This study also identifies the association between socioeconomic and demographic barriers with vitamin D supplementation among children. Children in high-income families (43.63%) were more likely to take vitamin D supplements than those in middle- or low-income families.

Parents with private health insurance (51.85%) were more likely to provide vitamin D supplements to their children compared to those with government health insurance (25.92%) and no health insurance (22.22%).

Furthermore, it was found in this study that parents’ education and literacy level largely determines children receiving vitamin D supplementation. Parents in this study with a lower literacy rate who did not complete primary school were less likely to give vitamin D supplements to their children. However, evidence of gender differentials in vitamin D supplementation was not found in this study.

In U.A.E., since there is no law requiring the fortification of vital foods with vitamin D, there are few vitamin D-fortified products on the market. ^26^ Among the participants of the study, 184 (74.2%) reported the child’s diet to contain multiple natural sources of vitamin D (for example some children had milk plus cheese in their diet while others had yogurt plus cheese plus vitamin D fortified orange juice) and most parents reported that their child has milk in their diet. Moreover, 69 parents (27.8%) reported giving none of the natural sources of vitamin D to their children through the diet. As a result, individual vitamin D dietary intake is strongly influenced by dietary preferences as well as the country's fortification plan. Without supplementation, however, vitamin D status is heavily on endogenous vitamin D synthesis, which is influenced by genetic determinants and lifestyle. ^27^

Vitamin D aids calcium absorption in the intestine by facilitating active calcium transport across the mucosa. Vitamin D insufficiency is usually caused by a lack of calcium in the diet and leads to bone deterioration or osteoporosis. ^28^ The results of this study are generalizable since it has been done on general population and multiple ethnicities in the U.A.E, it reflects that this study is generalizable to different parts of the world.

### Limitations of the study

However, this study had potential limitations. From a methodological point of view, the weakness of the study is that it is based on a cross-sectional design. The inherent problem of a cross-sectional design is that the outcome (vitamin D supplementation status) and the exposure (in this case, socioeconomic characteristics and a State’s social and economic development status) are collected simultaneously, thereby preventing conclusions regarding causality. The data was mostly collected from mothers. The present literature lacks data on the vitamin D level of children, thus a comparison between outdoor level activity of the children and the vitamin D level of children was presented in this study.

## Conclusion

The findings of the study concluded that the educational and financial background of parents and health insurance status could aid in addressing the challenges parents face with providing vitamin D supplements to their children as well as nutritional assessment for early natural supplement treatment.

## Data Availability

Figshare. Parents reported Vitamin D Supplementation among Children (Responses). DOI: https://doi.org/10.6084/m9.figshare.20207165.v1. ^29^ This project contains the following data:
- The purpose of this study was to review the vitamin D supplementation intake status among children in the general public, determine the vitamin D supplements practices, and the barriers that parents and children face with supplementation.

## References

[1] DeLuca HF : The metabolism and functions of vitamin D. *Steroid Hormone Resistance.* 1986; pp.361–375. 10.1007/978-1-4684-5101-6_24 3012979

[2] Bikle DD : Vitamin D and bone. *Curr. Osteoporos. Rep.* 2012 Jun;10(2):151–159. 10.1007/s11914-012-0098-z 22544628 PMC3688475

[3] Feng X McDonald JM : Disorders of bone remodeling. *Annual Review of Pathology: Mechanisms of Disease.* 2011 Feb 28;6:121–145. 10.1146/annurev-pathol-011110-130203 20936937 PMC3571087

[4] Fleet JC DeSmet M Johnson R : Vitamin D, and cancer: a review of molecular mechanisms. *Biochem. J.* 2012 Jan 1;441(1):61–76. 10.1042/BJ20110744 22168439 PMC4572477

[5] Bikle DD : Vitamin D: production, metabolism, and mechanisms of action. *Endotext.* 2021 Dec 31.

[6] Cashman KD : Vitamin D in childhood and adolescence. *Postgrad. Med. J.* 2007 Apr 1;83(978):230–235. 10.1136/pgmj.2006.052787 17403948 PMC2600028

[7] Weydert JA : Vitamin D in children’s health. *Children.* 2014 Sep;1(2):208–226. 10.3390/children1020208 27417476 PMC4928729

[8] Sahay M Sahay R : Rickets–vitamin D deficiency and dependency. *Indian J. Endocrinol. Metab.* 2012 Mar;16(2):164–176. 10.4103/2230-8210.93732 22470851 PMC3313732

[9] Kassai MS Cafeo FR Affonso-Kaufman FA : Vitamin D plasma concentrations in pregnant women and their preterm newborns. *BMC Pregnancy Childbirth.* 2018 Dec;18(1):1–8. 30348112 10.1186/s12884-018-2045-1 PMC6198501

[10] Battersby AJ Kampmann B Burl S : Vitamin D in early childhood and the effect on immunity to Mycobacterium tuberculosis. *Clin. Dev. Immunol.* 2012 Jan 1;2012. 10.1155/2012/430972 PMC3398646 22829851

[11] Lee JY So TY Thackray J : A review on vitamin d deficiency treatment in pediatric patients. *J. Pediatr. Pharmacol. Ther.* 2013 Oct;18(4):277–291. 10.5863/1551-6776-18.4.277 24719588 PMC3979050

[12] Gröber U Spitz J Reichrath J : Vitamin D: update 2013: from rickets prophylaxis to general preventive healthcare. *Dermato-endocrinology.* 2013 Jun 1;5(3):331–347. 10.4161/derm.26738 24516687 PMC3908963

[13] Lai YH Fang TC : The pleiotropic effect of vitamin D. *International Scholarly Research Notices.* 2013;2013.

[14] Dominguez LJ Farruggia M Veronese N : Vitamin D sources, metabolism, and deficiency: available compounds and guidelines for its treatment. *Metabolites.* 2021 Apr;11(4):255. 10.3390/metabo11040255 33924215 PMC8074587

[15] AlFaris NA AlKehayez NM AlMushawah FI : Vitamin D deficiency and associated risk factors in women from Riyadh, Saudi Arabia. *Sci. Rep.* 2019 Dec 30;9(1):1–8. 31889122 10.1038/s41598-019-56830-z PMC6937288

[16] Roseland JM Phillips KM Patterson KY : Feldman D Pike JW Bouillon R , editors. *Vitamin D in foods: An evolution of knowledge.* 41–78.

[17] Food US Administration D : Food additives permitted for direct addition to food for human consumption; vitamin D2 mushroom powder. *Fed. Regist.* 2020;85:41916–41920.

[18] U.S. Food and Drug Administration:January 4, 2018. Reference Source

[19] Muhairi SJ Mehairi AE Khouri AA : Vitamin D deficiency among healthy adolescents in al ain, united Arab emirates. *BMC Public Health.* 2013 Dec;13(1):1–7. 10.1186/1471-2458-13-33 23311702 PMC3610121

[20] Al-Othman A Al-Musharaf S Al-Daghri NM : Effect of physical activity and sun exposure on vitamin D status of Saudi children and adolescents. *BMC Pediatr.* 2012 Dec [cited 2021 Mar 11];12(1):589. 10.1186/1471-2431-12-92 22759399 PMC3407533

[21] Hirani V Mosdøl A Mishra G : Predictors of 25-hydroxyvitamin D status among adults in two British national surveys. *Br. J. Nutr.* 2008 Jul 17 [cited 2021 Mar 11];101(5):760–764. Reference Source 18631415 10.1017/S0007114508023416 PMC3491866

[22] Giovannucci E Liu Y Rimm EB : Prospective Study of Predictors of Vitamin D Status and Cancer Incidence and Mortality in Men. *JNCI J. Natl. Cancer Inst.* 2006 Apr 5 [cited 2021 Mar 11];98(7):451–459. 10.1093/jnci/djj101 Reference Source 16595781

[23] Nucci AM Russell CS Luo R : The effectiveness of a short food frequency questionnaire in determining vitamin D intake in children. *Dermato-endocrinology.* 2013 Jan 1;5(1):205–210. 10.4161/derm.24389 24494056 PMC3897592

[24] Reference Source

[25] Kull M Jr Kallikorm R Tamm A : Seasonal variance of 25-(OH) vitamin D in the general population of Estonia, a Northern European country. *BMC Public Health.* 2009;9:22. 10.1186/1471-2458-9-22 19152676 PMC2632995

[26] Hwalla N Al Dhaheri AS Radwan H : The prevalence of micronutrient deficiencies and inadequacies in the Middle East and approaches to interventions. *Nutrients.* 2017;9:229.28273802

[27] Wang TJ Zhang F Richards JB : Common genetic determinants of vitamin D insufficiency: a genome-wide association study. *Lancet.* 2010;376:180–188. 10.1016/S0140-6736(10)60588-0 20541252 PMC3086761

[28] Heaney RP : Vitamin D and calcium interactions: functional outcomes. *Am. J. Clin. Nutr.* 2008;88:541S–544S. 10.1093/ajcn/88.2.541S 18689398

[29] Sharafi N Fatima A Gillani SW : Parents reported Vitamin D Supplementation among Children (Responses). figshare.Dataset.2022. 10.6084/m9.figshare.20207165.v1

