# Evaluation of vitamin D supplementation intake among children; cross-sectional observational study

**DOI:** 10.12688/f1000research.123373.2

**Published:** 2023-03-13

**Authors:** Niloufar Sharafi, Aiman Fatima, Syed Wasif Gillani, Nour Kaddour, Rawa Banoori, Riham Mohamed Elshafie, Hassaan Anwer Rathore

**Affiliations:** 1 College of Pharmacy,, Gulf Medical University,, Ajman, United Arab Emirates; 2 Clinical and Hospital Pharmacy Department, College of Pharmacy, Taibah University, Al Madinah Al Munawwarah, Saudi Arabia; 3 Clinical Pharmacy Department, ASUSH,, Ain Shams University, Cairo, Egypt; 4 College of Pharmacy, QU Health, ,, Qatar University, Doha,, 2713,, Qatar

**Keywords:** Vitamin D, deficiency, frequency, supplementation, dietary sources, sunlight exposure, challenges

## Abstract

**Background:** The purpose of this study was to assess  the vitamin D supplementation intake status among children from different nationalities in the UAE,  to determine vitamin D intake practices through diet and lifestyle, and the barriers that parents in the UAE  face with providing vitamin D supplementation to their children.

**Methods:** A cross-sectional observational questionnaire-based survey study design was used.. The study was conducted in the U.A.E and the study participants were parents of children from ages 4-15 years. The questionnaire used in this study was both self-administered and interviewer-administered while inquiring the questions from the parents. A convenience sampling technique was used to collect the data. The response rate of participants was expected to be 63%, the margin of error was 5% and the level of confidence was 95%.

**Results:** A total of 248 participants (203 mothers, 39 fathers and 6 caregivers) completed the study. Participants reported that the supplements used the most by children were vitamin D supplements (21.85%), followed by multivitamins (21.8%) and calcium supplements (5.6%) and 27.8% of participants in this study reported  to  no  supplementation at all. The rate of vitamin D supplementation among children was higher in those families with higher income levels, parents/caregivers who were more educated, those families who attained health insurance. However, there was no statistical significance between these correlations.

**Conclusion:** The study concluded that challenges like the educational and financial background of parents, family-income level, and health insurance status could help aid in addressing the overall burden of vitamin D deficiency among young children in the UAE. Pediatricians and health care professionals could use our study and use it as an aid to provide screening on lifestyle, sun light exposure, and dietary modifications and also educate parents why and how vitamin D is crucial for their children.

## Introduction

Vitamin D is known among the critical minerals to play an important role in maintaining normal body functions. ^1^ It allows bone mineralization and avoids hypocalcemic tetany (such as involuntary muscle contraction, cramps, spasms, etc.). ^2^ It is also known for aiding osteoblasts and osteoclasts in developing and remodeling the bone preventing it from being brittle. ^3^ Other functions of vitamin D in the body include inflammation reduction and regulation of cell growth, neuromuscular and immune function, and glucose metabolism. ^4^ Vitamin D also affects the expression of several genes that code for proteins that govern cell proliferation, differentiation, and apoptosis. Vitamin D receptors can be found in many tissues, and some of them transform 25(OH) D to 1,25 (OH) D. ^5^ Vitamin D made in the skin or ingested in the diet is biologically inert and requires two successive hydroxylations first in the liver on carbon 25 to form 25-hydroxyvitamin D [25(OH)D], and then in the kidney for a hydroxylation on carbon 1 to form the biologically active form of vitamin D, 1,25-dihydroxyvitamin D [1,25(OH)2D]. ^5^

Maintaining optimum levels of calcium and vitamin D during childhood and adolescence is critical for bone growth. ^6^ Vitamin D is said to lower the risk of cancer, prevent viral infections, alleviate musculoskeletal pain, and calm mood disorders including depression, according to some claims. There has also been a surge in scientific interest in studying vitamin D at both the basic and clinical levels to address these and other claims. ^7^ Children with vitamin D deficiency develop a disease known as rickets, which is characterized by a frame and fragile bone, making the legs appear bent. ^8^ Vitamin D has been shown to reduce the risk of premature birth in pregnant women. ^9^

A child's vitamin D deficiency can start as early as birth, which can damage not just their bone metabolism but also their immunological system, making them more susceptible to illnesses early in life. ^10^ For the treatment of vitamin D deficiency rickets, the AAP recommends an initial 2- to 3-month regimen of “high-dose” vitamin D therapy of 1000 units daily in neonates, 1000 to 5000 units daily in infants 1 to 12 months old, and 5000 units daily in patients over 12 months old. ^11^

Epidemiologic studies, apart from the risk of osteomalacia and osteoporosis, have associated hypovitaminosis D with an increased risk of several cancers, autoimmune diseases (type 1 diabetes, multiple sclerosis, rheumatoid arthritis, and Crohn’s disease), heart disease, hypertension, metabolic syndrome, asthma, upper respiratory tract infections, muscle pain and weakness, and falling. It is estimated that the prevalence of deficiency is 62–95.7% in new-borns and breast-feeding groups (0–6 months), 46–80% in 6–60 months of age and 37.8–97.5% in 5–20-year-old children. ^12^ Vitamin D deficiency and insufficiency is linked with mood disorders and anxiety among youngsters and adults. The pleiotropic action of vitamin D was already revealed on molecular, cellular, tissue, and organ levels. ^13^ These observations modified the current knowledge about vitamin D metabolism and methods of diagnosis of vitamin D deficiency states. ^14^

Unfortunately, vitamin D is found rare in food. ^15^ Vitamin D is found in only a few foods. Fish liver oils and the meat of fatty fish (such as trout, salmon, tuna, and mackerel) are among the greatest sources. The amount of vitamin D in a human tissue is influenced by its food. Vitamin D is also found in modest levels in beef liver, egg yolks, and cheese, mostly in the form of vitamin D3 and its metabolite 25(OH)D3. ^16^ Vitamin D2 is found in varying levels in mushrooms. Some commercially available mushrooms have been exposed to UV radiation to boost their vitamin D2 levels. In addition, the FDA has approved UV-treated mushroom powder as a food additive for use as a vitamin D2 source in food items. ^17^

Furthermore, vitamin D is added to milk, many ready-to-eat bowls of cereal, and some yogurt and orange juice brands. It is found in modest concentrations in cheese and some margarine. ^18^

In the U.A.E., due to the consistently predominant hot weather, inadequate exposure to sunlight, and low nutritional intake of vitamin D, results in low serum concentrations of circulating 25(OH) D, a condition known as hypovitaminosis D among the general population. ^19^ Furthermore, recent lifestyles involving using cars for transport over walking, and children indulging in electronics and staying indoors have also influenced low vitamin levels. Low dietary intake of vitamin D and calcium, and other factors, including obesity and low social status, are all associated with low serum levels of vitamin D. ^20^ ^–^ ^22^

Further research is needed to be conducted on the production of high-potency–food-based vitamin D supplements, the move to mandatory fortification of cereal grain staples, and the development of natural food sources with higher vitamin D content. Although various studies suggest a high prevalence of vitamin D deficiency among adults and children, no randomized controlled trials have been fully performed on vitamin D deficiency and supplementation among children in the UAE. Consequently, the prevalence of vitamin D deficiency is high both in children and adults, and new guidelines are needed to overcome this major public health issue.

The purpose of this study was to assess the vitamin D supplementation intake status among children from different population subtypes in the UAE, the natural sources of vitamin D from their diet, and the barriers parents and children face with supplementation.

## Methods

### Study design and setting

A cross-sectional observational questionnaire-based survey study design was used in this study. The survey was conducted in public places like malls, parks and hospitals in the U.A.E. and the study participants were parents/caregivers of children from ages 4-to 15- years. The questionnaire was both self-administered and interviewer-administered. For parents/care givers who found it difficult to understand the questionnaire, the interviewer verbally inquired questions to the included participants individually from the questionnaire and filled the data on their behalves as they answered. The questionnaire was filled on a secure device with only the main investigators having access to it. The data was collected over a period of seven months from October 2021 to April 2022. Mean, standard deviation, and mean comparison was utilized for continuous data.

### Research tool

The questionnaire was adapted from another study; *The effectiveness of a short food frequency questionnaire in determining vitamin D intake in children* which was conducted on 296 healthy 6- to14-y-old African American and Caucasian children residing in Pittsburgh, Pennsylvania, USA and evaluated for content validation from the main authors of that study. ^23^ However, only a selected number of questions from the original questionnaire were used in the survey of our study. ^23^ Our survey questionnaire consisted of around 10 questions in total. ^23^ We had generated an Arabic and English version of it for participants’ ease.

Part one of the study questionnaire consists demographic information (e.g. age, nationality, gender, etc.). This part of the questionnaire also includes the literacy/education levels of the parents/caregivers, income levels and health insurance status of the parents/caregivers in the study. This part of the questionnaire comprises of the data on the barriers parents/caregivers face with providing vitamin D supplementation to their children.

In order to investigate the many possible reasons of vitamin D deficiency, the second part of our study questionnaire comprised of questions related to supplementation intake and natural sources of vitamin D intake through their diet (e.g, if the child has milk, yogurt, etc. in his diet). The last part consists questions on the outdoor activity level of the child (e.g. hours the child spends playing outdoors). Most parts of the data were collected based on yes or no, or multiple choice questions with a space for the ‘other’ responses as well. ^23^

### Variables

Vitamin D deficiency, education level of parents, outdoor activity hours of children, health insurance status of parents, income level of the families.

### Primary outcomes

Poor supplementation practices of vitamin D among children associated with low dietary sources of vitamin D, low exposure to sunlight, low literacy rates among parents/caregivers, low family income, absence of health insurance among the families are the primary outcomes of our study.

### Participants and sampling method

A convenient sampling technique was used to collect data from approximately 248 participants from public places in Ajman, U.A.E. An online Rao soft sample size calculator was applied to determine the sample size, which was 319. The response rate of parents was expected to be 63%, the margin of error was 5% and the level of confidence was 95%. ^24^ Around 319 participants were determined by the Rao soft sample size calculator, however 301 responded, 71 of them did not fit in our inclusion criteria and 248 participants responded and actively participated in the study, 77.7% was the response rate of the study participants.

### Missing data

This study has no missing data. A total of 248 participants were included and all 248 of them actively participated in our study. During analysis, we found no missing data on our participants.

### Inclusion criteria

Participants living in the UAE who had at least one child between 4-15 years of age and who agreed to participate.

### Exclusion criteria

Children with minor illnesses that are common in the general population and those suspected clinically of having rickets. Children with cognitive and behavioral disorders were excluded from the study to avoid bias which would affect the results.

### Ethical issues

Ethics approvals have been obtained for the study. This is the ethics approval number IRB/COP/STD/74/Oct-2021 from Gulf Medical University, Ajman.

### Consent form

The questionnaire content was described before letting the participants administer the data into it and the written consent form was taken from each participant prior to interviewing/handing out the questionnaire. The consent form was as follows:

“Your participation in this survey is voluntary. You may choose not to participate. If you decide to participate in this survey, you may withdraw at any time. If you decide not to participate in this study, or if you withdraw from participating at any time you will not be penalized. Filling out this form means that you accept to participate in this research.”

### Statistical analysis

The data analysis was done using the Statistical Package for Social Sciences (SPSS). Prevalence of low vitamin D supplementation and its distribution with socio-economic characteristic was analyzed. A Chi-square analysis was done. Mean, standard deviation, and mean comparison was utilized for continuous data. Both a tabular and graphic version of the data was used to show it. A 5% degree of confidence and a 0.5 margin of error were chosen.

### Bias

There is no bias in any trend in the collection, analysis, interpretation, or review of the data that can lead to conclusions that are different from the truth. However, since this was an observational study, variations in the results may be due to sample size.

## Results

A total of 248 participants; 81.9 % mothers (n=203) , 15.7% fathers (n=39) with a mean±SD age of 35.4±7.04 years, and 2.4% others who were guardians or care takers (n=6), completed the study. From our analysis on the socio-economic status of our participants, we found that about 62.9% (n=156) of our study participants had university level of education, while 26.2% (n=65) of our participants completed secondary school only, 9.7% (n=24) of participants only completed primary school as their level of education and 1.2% (n=3) of participants were uneducated.

Moreover, 61.7% (n=153) of mothers were unemployed, while a smaller percentage of mothers which is 27% (n=67) were employed, and 11.3% (n=28) of them were employed in a medical setting. As for the fathers who took part in our study, 70.2% (n=174) of them were employed and 21% (n=52), 4.8% (n=12) and 4% (n=10) were self-employed, employed in a medical setting, and unemployed, respectively. Approximately 42.7% (n=106) participants had a family income level of more than 10,000, while 35.5% (n=88), 10.1% (n=25) and 4.8% (n=12) of the families’ income levels were 5,000-10,000, 2,000-5,000 and less than 2,000, respectively. More than half of our study participants had a health insurance plan and only 28.6% (n=71) of our participants reported to have no health coverage ( Table 1).

**Figure:**
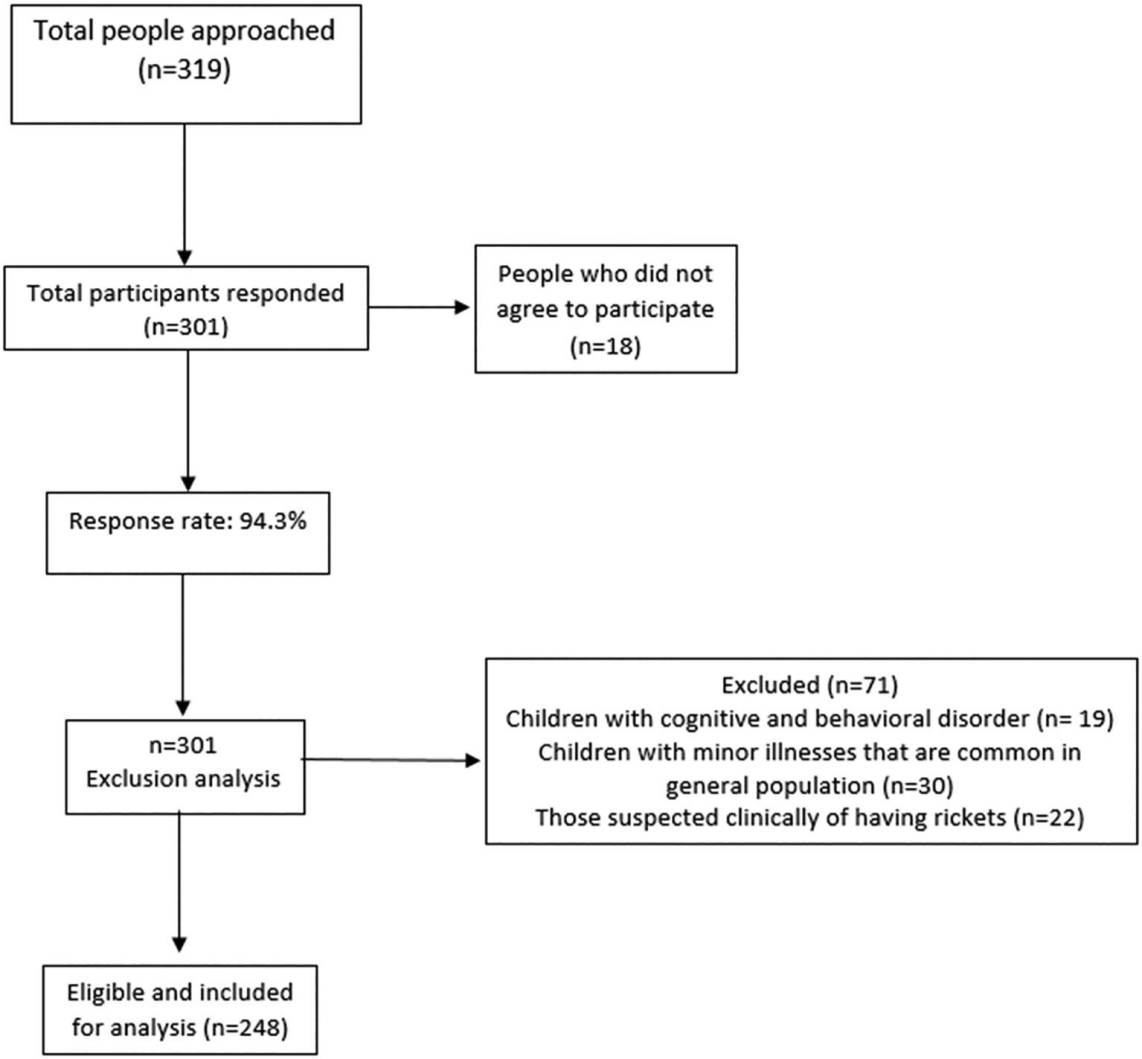
Study flow diagram.

**Table 1.** Sociodemographic parameters of the study participants.

Characteristics	N(%)
Gender	
Mother	203(81.9)
Father	39(15.7)
Others *	6(2.4)
Age (mean±S.D.)	35.4±7.042
Education Level	
Not educated	3(1.2)
Primary School	24(9.7)
Secondary School	65(26.2)
University	156(62.9)
Father Employment	
Employed	174(70.2)
Unemployed	10(4.0)
Self-employed	52(21)
Employed with Medical Background	12(4.8)
Mother Employment	
Employed	67(27)
Unemployed	153(61.7)
Employed with Medical Background	28(11.3)
Income	
Level Less than 2,000	12(4.8)
2,000-5,000	25(10.1)
5,000-10,000	88(35.5)
More than 10,000	106(42.7)
Insurance	
Government	51(20.6)
Private	126(50.8)
None	71(28.6)

* Caregivers.

**Figure 1.**
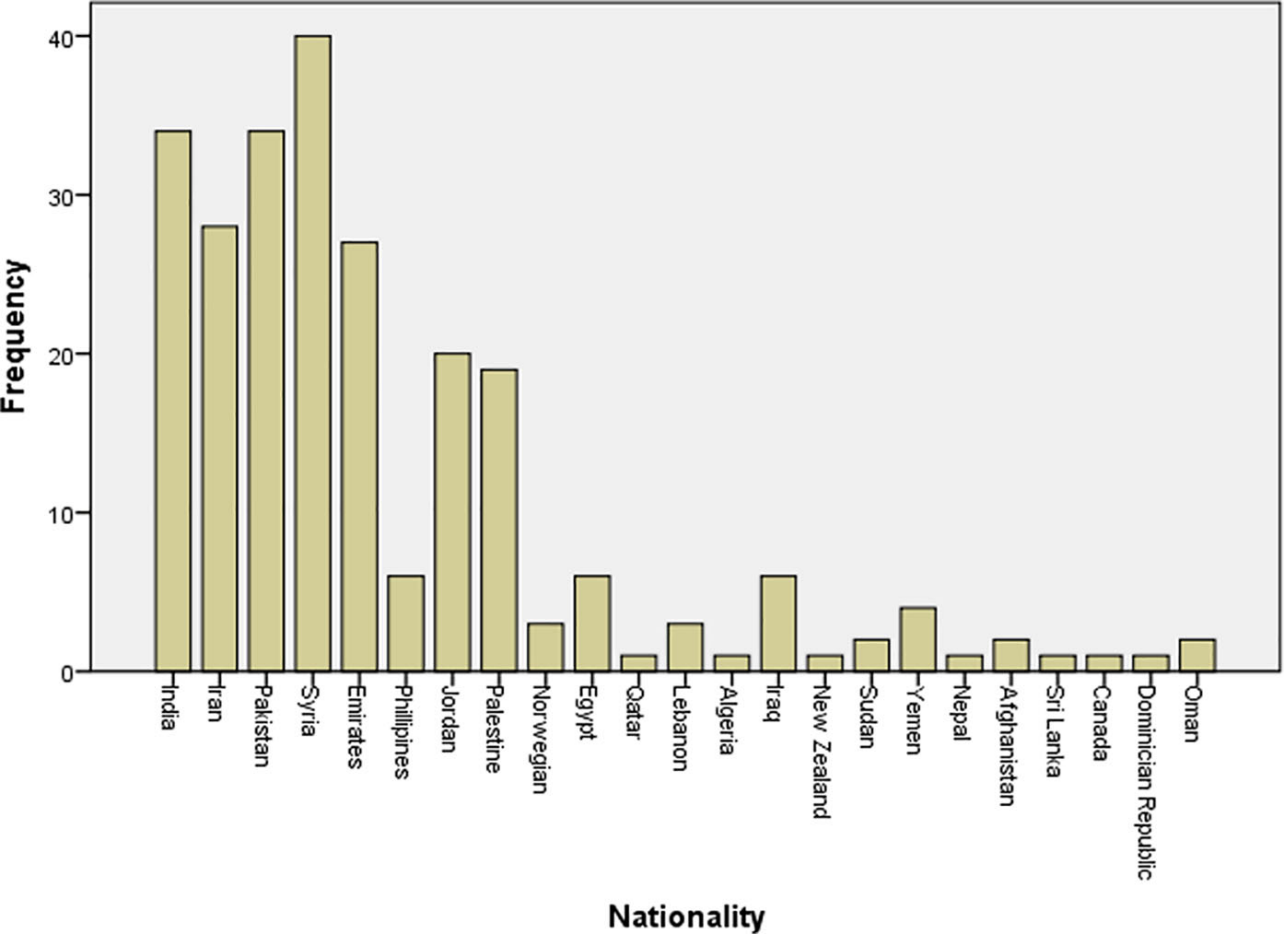
Nationality distribution of study participants.

This research has participants from different countries (total=23). The majority (67%) of the study participants are from five countries - India, Iran, Pakistan, Syria, and Emirates. The complete data has been presented in Figure 1.

A higher proportion of children received supplements is associated with parents/caregivers educated to the level of secondary school and above. Table 2 shows the literacy level of parents and supplementation.

**Table 2.** Literacy level of participants and supplementation practice.

		Supplements taken			
		Vitamin D	Calcium supplements	Multivitamins	Multiple sources
		N(%)	N(%)	N(%)	N(%)
Education level	Not educated	1(1.85)	1(7.14)	1(1.85)	0
	Primary school	5(9.25)	0	11(20.37)	3(5.35)
	Secondary school	15(27.77)	9(64.28)	10(18.51)	18(32.14)
	University	33(61.11)	4(28.57)	32(59.25)	35(62.5)

The data on outdoor activity levels included the average frequency of outdoor activity of a child per day. Of all the participants 65% (162) of them reported sending their child outside to play while 34.67% (86) reported no outdoor activity. The mean hours of outdoor activity for the children were 2.046 ±1.61. It was found that on average, children who are active outdoors, spent 0.15-6 hours playing outside in the sun thereby being exposed to sunlight ( Table 3).

**Table 3.** Outdoor activity level of children.

Characteristics	N(%)
Activity	
Yes	162(65)
No	86(34.67)
Hours mean±S.D.	2.046±1.61
Min-Max (hours)	0.15-6 hours

**Figure 2.**
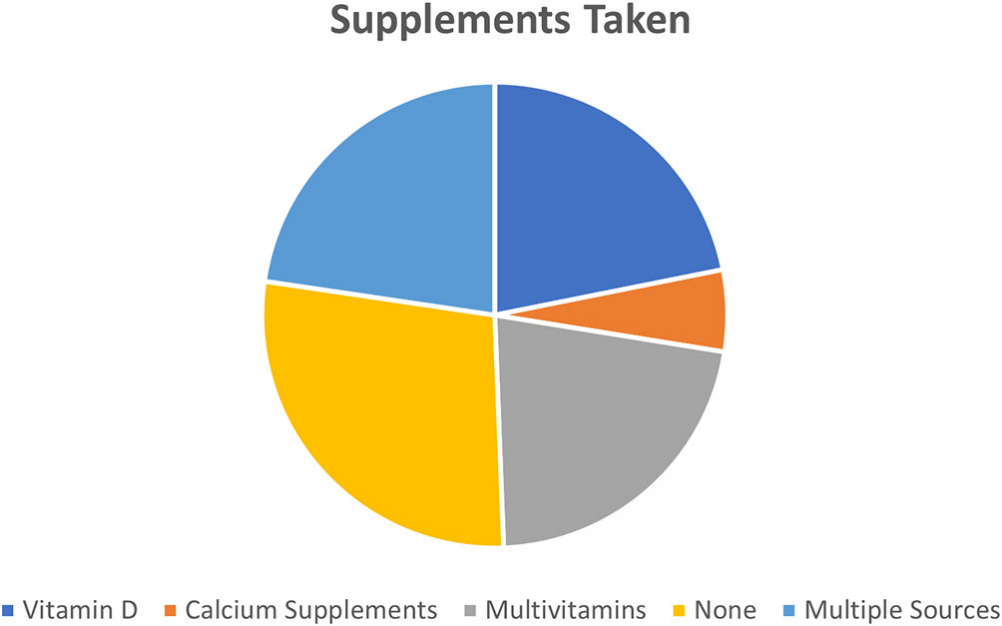
Supplementation intake among the study participants.

Participants reported that supplements used the most by children were Vitamin D supplements (21.85%) and multivitamins (21.8%) followed by calcium supplements (5.6%). However, 27.8% of children in this study did not take any supplements. While other parents/caregivers reported mixed intake of supplements (vitamin D plus calcium supplements or vitamin D and multivitamins) by their children ( Figure 2).

**Figure 3.**
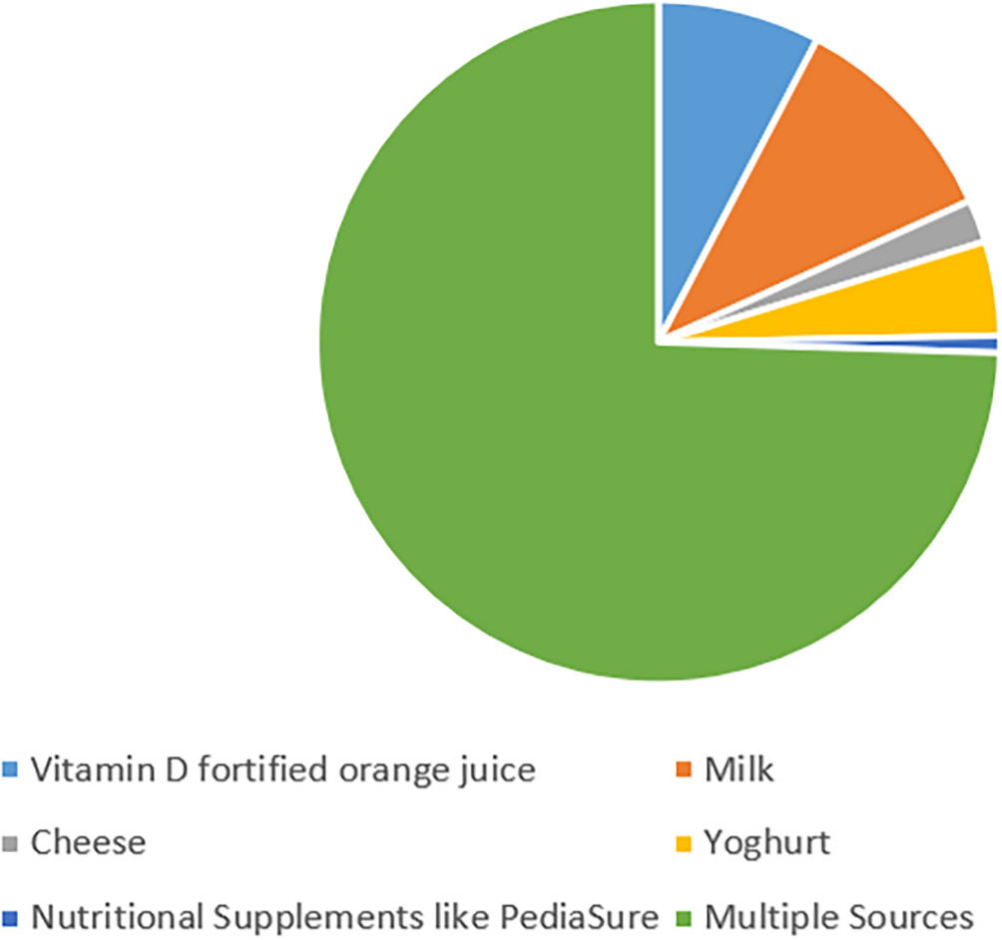
Children’s intake of natural sources containing vitamin D.

Figure 3 summarizes and describes that out of the 248 participants, 184 (74.2%) parents reported their children’s diet to contain multiple natural sources of vitamin D (milk plus cheese, or yoghurt plus cheese plus vitamin D fortified orange juice). However, 69 (27.8%) of our study participants reported giving no sources of natural vitamin D to their children through diet.

Children belonging to high-income families (43.63%) were more likely to receive vitamin D supplements than those in middle- or low-income families ( Table 4).

**Table 4.** Participant’s income level and supplementation practices.

		Supplements taken				
		Vitamin D	Calcium supplements	Multivitamins	Multiple sources	None
		N(%)	N(%)	N(%)	N(%)	N(%)
Income level	Less than 2,000	5(9.09)	0	6(11.11)	1(1.78)	2(2.89)
	2,000-5,000	8(14.54)	0	8(14.81)	6(10.71)	8(11.59)
	5,000-10,000	18(32.72)	7(50)	17(31.48)	21(37.5)	30(43.47)
	More than 10,000	24(43.63)	7(50)	23(42.59)	28(50)	29(42.02)

Parents/caregivers with private health insurance (51.85%) were more likely to provide vitamin D supplements to their children compared to those with government health insurance (25.92%) and no health insurance (22.22%) ( Table 5).

**Table 5.** Health insurance status of study participants and vitamin D supplementation intake.

		Supplements taken			
		Vitamin D	Calcium supplements	Multivitamins	Multiple sources
		N(%)	N(%)	N(%)	N(%)
Insurance	Governmental	14(25.92)	0	14(25.92)	5(8.92)
	Private	28(51.85)	14(100)	20(37.03)	38(67.85)
	No insurance	12(22.22)	0	20(37.03)	13(23.21)

Our study included participants from 23 various nationalities. The top five nationalities with the most number of participants were, India, Pakistan, Syria, and the U.A.E. Out of 34 participants from India, only 11 (32.35%) reported the use of vitamin D supplementation by their children, four (11.76%) reported using calcium supplements, six (17.64%) reported using multivitamins and the other 6 of them (17.64%) reported giving multiple sources of vitamins to their children.

However, out of the 34 participants from India, seven (20.58%) did not use any vitamin supplements at all. While out of 28 participants from Iran, only 6 participants, (21.41%) reported the use of vitamin D supplementation by their children, three (10.71%) reported using calcium supplements, ten (35.71%) reported using multivitamins and the other eight of them (28.57%) reported giving multi-sources of vitamins to their children. Among the 34 participants from Pakistan, the lowest amount of vitamin D supplements intake, only four (11.76%) participants reported giving supplements to their children and three (8.82%) reported using calcium supplements, only one (2.94%) parent reported using multivitamins and the other 12 of them (34.29%) reported giving multi-sources of vitamins to their children. Out of 27 participants from Emirates, few participants which are five (18.51%) of them reported the use of vitamin D supplementation by their children, two (7.4%) reported using calcium supplements, seven (25.94%) reported using multivitamins and the rest 11 (40.74%) reported giving multi-sources of vitamins to their children. The participants from Syria reported the highest vitamin D intake among the countries, out of 40 participants from India, only 12 (30.76%) reported the use of vitamin D supplementation by their children, one (2.56%) reported using calcium supplements, nine (23.07%) reported using multivitamins and the other 10 (25.64%) reported giving multiple sources of vitamins to their children ( Table 6).

**Table 6.** Nationality of study participants [top 5=163 (67%)] and vitamin supplements.

	Supplements taken				
	Vitamin D	Calcium supplements	Multivitamins	Multiple sources	None
	N(%)	N(%)	N(%)	N(%)	N(%)
India (N=34)	11(32.35)	4(11.76)	6(17.64)	6(17.64)	7(20.58)
Iran (N=28)	6(21.42)	3(10.71)	10(35.71)	1(3.57)	8(28.57)
Pakistan (N=34)	4(11.76)	3(8.82)	1(2.94)	12(34.29)	14(41.17)
Syria (N=40)	12(30.76)	1(2.56)	9(23.07)	10(25.64)	7(17.94)
Emirates (N=27)	5(18.51)	2(7.4)	7(25.92)	11(40.74)	2(7.4)

The association between low vitamin D supplementation and socio-economic and demographic characteristics, exposure to sunlight, dietary sources of vitamin D, health insurance status was assessed and the significance of these associations was tested using chi square test. We could not observe any significant associations with low vitamin D supplementation and the other factors.

## Discussion

Several studies have reported the association between socio-demographic and economic factors among parents/caregivers and low vitamin D supplementation practices. ^25^ There are no previous studies on the prevalence of low vitamin D supplementation practices in children living in the UAE. This cross-sectional study identifies the association between socioeconomic and demographic factors and vitamin D supplementation practices among children. It is noted from our study that due to the consistent hot climate in the UAE, children get little exposure to sunlight. Dietary sources of vitamin D also perhaps plays a vital role in stimulating vitamin D production among young and growing children. ^26^

It was found in this study that higher literacy rates among parents, higher income levels and health insurance in the families, increases the frequency of a child’s intake of vitamin D supplementation.

In U.A.E., since there is no law requiring the fortification of vital foods with vitamin D, there are a few vitamin D-fortified products in the market. ^27^ Among the participants of our study, 184 (74.2%) reported the child’s diet to contain multiple natural sources of vitamin D. However, 69 parents (27.8%) reported giving none of the natural sources of vitamin D to their children through the diet.

As a result, individual vitamin D dietary intake is strongly influenced by dietary preferences as well as the country's fortification plan. Without supplementation, vitamin D status is heavily on endogenous vitamin D synthesis, which is influenced by genetic determinants and lifestyle. ^28^ It would be useful to have national prevalence data on vitamin D deficiency in the UAE, because in addition to its impact on calcium homeostasis, there is accumulating evidence that vitamin D also plays a role in avoiding the onset of diabetes mellitus and numerous other health-related diseases in children.

Vitamin D aids calcium absorption in the intestine by facilitating active calcium transport across the mucosa. Vitamin D insufficiency is usually caused by a lack of calcium in the diet and leads to bone deterioration or osteoporosis. ^29^ The results of this study are generalizable since it has been conducted on general population of children from multiple ethnicities in the U.A.E.

### Limitations of the study

However, this study had potential limitations. From a methodological point of view, the weakness of the study is that it is based on a cross-sectional design. The inherent problem of a cross-sectional design is that the outcome (vitamin D supplementation status) and the exposure (in this case, socioeconomic characteristics) are collected simultaneously, thereby preventing conclusions regarding causality. The data was mostly collected from mothers giving rise to culture or gender bias. A convenient sampling technique was used to collect data from approximately 248 participants. The potential drawback of convenient sampling technique is:

• Sampling bias
• Selection bias
• Difficult to generalize data
• Breaking down of results into demographic data would be difficult

## 5. Conclusion

The epidemiological findings of this study signify the reasons of prevalence of vitamin D deficiency among children from different population subtypes in the UAE. Vitamin D being a crucial element in the growth, development and immunity of children, its deficiency poses a great public health challenge. From our study we understand that factors such as low sun-light exposure, low dietary intake of vitamin D sources, and low intake of vitamin D supplements are the fundamental causes of vitamin D deficiency. Other reasons such as low monthly income of the families, low literacy rates among parents and no health insurance can have an impact on vitamin D supplementation practices. Pediatricians, health care professionals, and ministry of health could use this study as an aid to provide screening on lifestyle, sun light exposure, and dietary modifications, provide access to vitamin D and other supplementation to children and also educate parents why and how vitamin D is crucial for young children.

## Data availability

Parents reported Vitamin D Supplementation among Children (Responses). DOI: https://doi.org/10.6084/m9.figshare.20207165.v1. ^30^

This project contains the following data:

- The purpose of this study was to assess the vitamin D supplementation intake status among children in the general public, determine the vitamin D supplements practices, and the barriers that parents and children face with supplementation.

Data are available under the terms of the Creative Commons Attribution 4.0 International License (CC BY 4.0)

## References

[1] DeLuca HF : The metabolism and functions of vitamin D. *Steroid Hormone Resistance.* 1986; pp.361–375. 10.1007/978-1-4684-5101-6_24 3012979

[2] Bikle DD : Vitamin D and bone. *Curr. Osteoporos. Rep.* 2012 Jun;10(2):151–159. 10.1007/s11914-012-0098-z 22544628 PMC3688475

[3] Feng X McDonald JM : Disorders of bone remodeling. *Annual Review of Pathology: Mechanisms of Disease.* 2011 Feb 28;6:121–145. 10.1146/annurev-pathol-011110-130203 20936937 PMC3571087

[4] Fleet JC DeSmet M Johnson R : Vitamin D, and cancer: a review of molecular mechanisms. *Biochem. J.* 2012 Jan 1;441(1):61–76. 10.1042/BJ20110744 22168439 PMC4572477

[5] Bikle DD : Vitamin D: production, metabolism, and mechanisms of action. *Endotext.* 2021 Dec 31.

[6] Cashman KD : Vitamin D in childhood and adolescence. *Postgrad. Med. J.* 2007 Apr 1;83(978):230–235. 10.1136/pgmj.2006.052787 17403948 PMC2600028

[7] Weydert JA : Vitamin D in children’s health. *Children.* 2014 Sep;1(2):208–226. 10.3390/children1020208 27417476 PMC4928729

[8] Sahay M Sahay R : Rickets–vitamin D deficiency and dependency. *Indian J. Endocrinol. Metab.* 2012 Mar;16(2):164–176. 10.4103/2230-8210.93732 22470851 PMC3313732

[9] Kassai MS Cafeo FR Affonso-Kaufman FA : Vitamin D plasma concentrations in pregnant women and their preterm newborns. *BMC Pregnancy Childbirth.* 2018 Dec;18(1):1–8. 30348112 10.1186/s12884-018-2045-1 PMC6198501

[10] Battersby AJ Kampmann B Burl S : Vitamin D in early childhood and the effect on immunity to Mycobacterium tuberculosis. *Clin. Dev. Immunol.* 2012 Jan 1;2012. 10.1155/2012/430972 PMC3398646 22829851

[11] Lee JY So TY Thackray J : A review on vitamin d deficiency treatment in pediatric patients. *J. Pediatr. Pharmacol. Ther.* 2013 Oct;18(4):277–291. 10.5863/1551-6776-18.4.277 24719588 PMC3979050

[12] Surve S Chauhan S Amdekar Y : Vitamin D deficiency in children: An update on its prevalence, therapeutics and knowledge gaps. *Indian J. Nutr.* 2017;4(3):167.

[13] Lai YH Fang TC : The pleiotropic effect of vitamin D. *International Scholarly Research Notices.* 2013;2013.

[14] Dominguez LJ Farruggia M Veronese N : Vitamin D sources, metabolism, and deficiency: available compounds and guidelines for its treatment. *Metabolites.* 2021 Apr;11(4):255. 10.3390/metabo11040255 33924215 PMC8074587

[15] AlFaris NA AlKehayez NM AlMushawah FI : Vitamin D deficiency and associated risk factors in women from Riyadh, Saudi Arabia. *Sci. Rep.* 2019 Dec 30;9(1):1–8. 31889122 10.1038/s41598-019-56830-z PMC6937288

[16] Roseland JM Phillips KM Patterson KY : Feldman D Pike JW Bouillon R , editors. *Vitamin D in foods: An evolution of knowledge.* 41–78.

[17] Food US Administration D : Food additives permitted for direct addition to food for human consumption; vitamin D2 mushroom powder. *Fed. Regist.* 2020;85:41916–41920.

[18] Center for Food Safety and Applied Nutrition : Changes to the nutrition facts label [Internet]. U.S. Food and Drug Administration. FDA; [cited2023Jan30]. https://www.fda.gov/food/food-labeling-nutrition/changes-nutrition-facts-label

[19] Muhairi SJ Mehairi AE Khouri AA : Vitamin D deficiency among healthy adolescents in al ain, united Arab emirates. *BMC Public Health.* 2013 Dec;13(1):1–7. 10.1186/1471-2458-13-33 23311702 PMC3610121

[20] Al-Othman A Al-Musharaf S Al-Daghri NM : Effect of physical activity and sun exposure on vitamin D status of Saudi children and adolescents. *BMC Pediatr.* 2012 Dec [cited 2021 Mar 11];12(1):589. 10.1186/1471-2431-12-92 22759399 PMC3407533

[21] Hirani V Mosdøl A Mishra G : Predictors of 25-hydroxyvitamin D status among adults in two British national surveys. *Br. J. Nutr.* 2008;101(5):760–764. 10.1017/S0007114508023416 18631415 PMC3491866

[22] Giovannucci E Liu Y Rimm EB : Prospective Study of Predictors of Vitamin D Status and Cancer Incidence and Mortality in Men. *J. Natl. Cancer Inst.* 5 April 2006;98(7):451–459. 10.1093/jnci/djj101 16595781

[23] Nucci AM Russell CS Luo R : The effectiveness of a short food frequency questionnaire in determining vitamin D intake in children. *Dermato-endocrinology.* 2013 Jan 1;5(1):205–210. 10.4161/derm.24389 24494056 PMC3897592

[24] Sample size calculator [Internet] . Raosoft, Inc. makes high quality web survey software. http://www.raosoft.com/samplesize.html

[25] Hurmuzlu Kozler S Saylı TR : Factors influencing initiation and discontinuation of vitamin D supplementation among children 1-24-months-old. *Curr. Med. Res. Opin.* 2022 Mar 4;38(3):435–441. 34817302 10.1080/03007995.2021.2010460

[26] Kull M Jr Kallikorm R Tamm A : Seasonal variance of 25-(OH) vitamin D in the general population of Estonia, a Northern European country. *BMC Public Health.* 2009;9:22. 10.1186/1471-2458-9-22 19152676 PMC2632995

[27] Hwalla N Al Dhaheri AS Radwan H : The prevalence of micronutrient deficiencies and inadequacies in the Middle East and approaches to interventions. *Nutrients.* 2017;9:229.28273802

[28] Wang TJ Zhang F Richards JB : Common genetic determinants of vitamin D insufficiency: a genome-wide association study. *Lancet.* 2010;376:180–188. 10.1016/S0140-6736(10)60588-0 20541252 PMC3086761

[29] Heaney RP : Vitamin D and calcium interactions: functional outcomes. *Am. J. Clin. Nutr.* 2008;88:541S–544S. 10.1093/ajcn/88.2.541S 18689398

[30] Sharafi N Fatima A Gillani SW : Parents reported Vitamin D Supplementation among Children (Responses). figshare.Dataset.2022. 10.6084/m9.figshare.20207165.v1

