# Supplementary material for: Evaluation of vitamin D supplementation intake among children; cross-sectional observational study
Source: F1000Res. 2023 Mar 13;11:1456. Originally published 2022 Dec 8. [Version 2] doi: 10.12688/f1000research.123373.2 (PMC10028306; doi:10.12688/f1000research.123373.2)
Supplement: Peer review history

# Peer review: Reviewer response for version 2

**DOI:** 10.5256/f1000research.144822.r166240

**Published:** 2023-03-20

**Authors:** Muhammad Umair Khan

**Affiliations:** 1 Aston Pharmacy School, College of Health and Life Sciences, Aston University, Birmingham, UK

I have no further comments

Is the work clearly and accurately presented and does it cite the current literature?

Partly

If applicable, is the statistical analysis and its interpretation appropriate?

I cannot comment. A qualified statistician is required.

Are all the source data underlying the results available to ensure full reproducibility?

Yes

Is the study design appropriate and is the work technically sound?

Partly

Are the conclusions drawn adequately supported by the results?

Partly

Are sufficient details of methods and analysis provided to allow replication by others?

Partly

Reviewer Expertise:

Medicine optimisation, mental health

I confirm that I have read this submission and believe that I have an appropriate level of expertise to confirm that it is of an acceptable scientific standard.

# Peer review: Reviewer response for version 2

**DOI:** 10.5256/f1000research.144822.r166239

**Published:** 2023-03-14

**Authors:** Tauqeer Hussain Mallhi

**Affiliations:** 1 Department of Clinical Pharmacy, College of Pharmacy, Jouf University, Al-Jouf, Saudi Arabia

The authors have addressed all my comments. I have no further queries related to this manuscript. Thank you for considering the suggestions.

Is the work clearly and accurately presented and does it cite the current literature?

Yes

If applicable, is the statistical analysis and its interpretation appropriate?

Yes

Are all the source data underlying the results available to ensure full reproducibility?

Yes

Is the study design appropriate and is the work technically sound?

Yes

Are the conclusions drawn adequately supported by the results?

Yes

Are sufficient details of methods and analysis provided to allow replication by others?

Yes

Reviewer Expertise:

Pharmacy Practice, Clinical Pharmacy, Pharmacotherapy

I confirm that I have read this submission and believe that I have an appropriate level of expertise to confirm that it is of an acceptable scientific standard.

# Peer review: Reviewer response for version 1

**DOI:** 10.5256/f1000research.135473.r157815

**Published:** 2022-12-19

**Authors:** Muhammad Umair Khan

**Affiliations:** 1 Aston Pharmacy School, College of Health and Life Sciences, Aston University, Birmingham, UK

This study explores an important subject on vitamin D supplementation among children in the United Arab Emirates. However, the manuscript needs significant revision before it can be published. I have raised some questions/comments in each section which authors may consider in preparing their revisions.  

**Abstract**

- Background
  - I suggest using ‘investigate’ or ‘assess’ rather than review.
  - Authors can avoid ‘general public’. ‘among children’ is just fine.
- Methods
  - Consider adding information like: who were the participants? Where was the study conducted? Any statistics used?
- Results
  - As per the information provided, 89.1% were mothers and 15.7% were fathers. It gives a total of 104.8%. Please check these numbers again.
- Conclusion
  - Please reconsider the conclusion. It is not clear what the authors are trying to suggest.

**Introduction**

- 1 ^st^ paragraph – last sentence – for the ease of readers, please explain what happens when 25(OH) D transforms to 1,25 (OH) D.
- 3 ^rd^ paragraph – last sentence - 5000 units daily in patients over 12 months old – is there any upper age limit and it applies to all including adults?
- 4 ^th^ paragraph - What about epidemiological studies in children? Are there any? What do they say? In my opinion, it is important to answer these questions as the population of interest in this is children.
-  7 ^th^ paragraph – first sentence – in children, adults, or general population?
- 8 ^th^ paragraph – first sentence – too long to comprehend. Please rewrite to improve clarification.
- 8 ^th^ paragraph – second sentence - What is the prevalence of vitamin D deficiency among adults and children globally and in the UAE?
- 8 ^th^ paragraph – second sentence - Are authors trying to imply that RCTs are the most appropriate research designs to determine prevalence? Please reconsider and rewrite this sentence.
- 9 ^th^ paragraph – I would suggest write a broader aim and then the objectives that this study tried to achieve.
- Also, the phrase ‘among children in the general public’ sounds a bit odd to me. I think ‘among children’ in just fine.

**Methods**

- Can authors provide some examples of the public places that were used?
- Can authors provide some references from where the questionnaire was adapted? Where were those studies conducted? Are there any cultural differences? How did the authors account for that?
- How many questions were there in total and in each section?
- Which section had yes/no and multiple response questions? Were there mixed question in each section?
- How did the authors evaluate the barriers to supplementation? The three sections do not tell anything about the barriers.
- How did the authors investigate the reasons for vitamin D deficiency? I am not sure it was mentioned earlier until this point.
- I am not sure what the authors are trying to tell through the primary outcomes. The first two points look like the objectives. The third point looks like a hypothesis, but I am not sure what that means.
- Around 319 participants were expected – Are the authors trying to imply that 319 participants were contacted?
- Response rate is excellent. How do the authors compare this with the similar other studies?
- Missing data – surprising!
- I suggest authors should mention earlier in the methods section that parents completed the survey.
- Whether the questionnaire was self-administered or interviewer-administered?
- Looking at the information presented in ‘consent form’, it appears that the questionnaire was self-administered. Did the parents completed the questionnaire right there and then? How did they return the questionnaire?
- Why the authors excluded children with minor illnesses as well as those with cognitive impairment? It was parents who completed the survey.
- A chi-square analysis was done to ………..?
- Both a tabular and graphic version of the data was used to show it – generally we use one form of representation to avoid duplication.
- Bias – Given this is an observational study, how can the authors assure this?
- Authors did not mention about the language in which the questionnaire was designed and distributed to the participants.

**Results**

- 1 ^st^ sentence – It was mentioned earlier. Avoid duplication.
- 2 ^nd^ sentence – I think the brackets are not utilised appropriately.
- From what I understand, 203 mothers and 39 fathers participated in the study. It makes a total of 242 participants. What about other 6 participants?
- If 203 is the correct number, it is 81.8% of the total participants not 89.1%.
- As per the information provided, 89.1% were mothers and 15.7% were fathers. It gives a total of 104.8%. Please check these numbers again.
- I strongly suggest authors to review their calculations and rewrite the results section.    
- The name of countries in Fig 1 was not readable.
- It was mentioned earlier that chi-square test was used but the results section did not mention chi-square values and any significance values.

 

**Discussion**

- The first 3 paragraphs of the discussion are simply results. I suggest authors to summarise the key results in the first paragraph to avoid duplication.  
- I think the discussion section needs considerable revision. Currently, it seems like a results section. I suggest authors to focus more explaining the results in light of the broader literature on the topic.
- The results of this study are generalizable – this statement needs more clarification. Earlier authors discussed the climate issues is UAE which is a contributing factor. Is the climate same in different parts of the world? Europe/US/Australia/Africa? Is not, how will the authors support their statement on generalisability?    

**Conclusion**

- I suggest authors to reconsider their conclusion. I am not sure what authors are trying to imply by saying that financial background of parents can aid in addressing the challenges of vitamin D supplementation.

Is the work clearly and accurately presented and does it cite the current literature?

Partly

If applicable, is the statistical analysis and its interpretation appropriate?

I cannot comment. A qualified statistician is required.

Are all the source data underlying the results available to ensure full reproducibility?

Yes

Is the study design appropriate and is the work technically sound?

Partly

Are the conclusions drawn adequately supported by the results?

Partly

Are sufficient details of methods and analysis provided to allow replication by others?

Partly

Reviewer Expertise:

Medicine optimisation, mental health

I confirm that I have read this submission and believe that I have an appropriate level of expertise to confirm that it is of an acceptable scientific standard, however I have significant reservations, as outlined above.

# Response

**Published:** 2023-02-23

**Authors:** Syed Wasif Gillani

**Affiliations:** Gulf Medical University, United Arab Emirates

**Comment 1:**

*Background*

*I suggest using ‘investigate’ or ‘assess’ rather than review.*

*Authors can avoid ‘general public’. ‘among children’ is just fine*

 Response 1: We agree with this comment therefore we have changed the word ‘review’ to ‘assess’.

 We also agree to omit ‘general public’ and mention ‘among children’.

**Comment 2:**

*Methods*

*Consider adding information like: who were the participants? Where was the study conducted? Any statistics used?*

 Response 2: Thank you for this suggestion. We have added better information for the method section which is now clearer. We have clarified the survey was conducted in public places like malls, parks and hospitals in the U.A.E and the study participants were parents/caregivers of children from ages 4-to 15- years. The questionnaire was both self-administered and interviewer-administered. For parents/care givers who found it difficult to understand the questionnaire, the interviewer verbally inquired questions to the included participants individually from the questionnaire and filled the data on their behalves as they answered. The questionnaire was filled on a secure device with only the main investigators having access to it.

**Comment 3:**

*Results*

*As per the information provided, 89.1% were mothers and 15.7% were fathers. It gives a total of 104.8%. Please check these numbers again.*

 Response 3:  We agree with the reviewer’s comment. We have recalculated our values and changed the numbers to the right ones.

**Comment 4:**

*Conclusion*

*Please reconsider the conclusion. It is not clear what the authors are trying to suggest.*

 Response 4: As suggested by the reviewer we have rephrased the conclusion to clearer one in the manuscript.

**Comment 5:**

*1 paragraph – last sentence – for the ease of readers, please explain what happens when 25(OH) D transforms to 1,25 (OH) D*

*Response 5:  *We agree to the reviewer’s comment and we have added information for the reader to understand what happens when 25(OH) D transforms to 1,25 (OH) D

**Comment 6**

*3 paragraph – last sentence - 5000 units daily in patients over 12 months old – is there any upper age limit and it applies to all including adults?*

*Response 6:  *We agree with this suggestion from the reviewer. Therefore, we have mentioned that for the treatment of vitamin D deficiency rickets, the AAP recommends an initial 2- to 3-month regimen of “high-dose” vitamin D therapy of 1000 units daily in neonates, 1000 to 5000 units daily in infants 1 to 12 months old, and 5000 units daily in patients over 12 months old.

**Comment 7**

*4 paragraph - What about epidemiological studies in children? Are there any? What do they say? In my opinion, it is important to answer these questions as the population of interest in this is children.*

*Response 7:  *Thank you for this suggestion. We agree to add some information on epidemiological studies in children. Therefore, we have mentioned that it is estimated that the prevalence of deficiency is 62–95.7% in new-borns and breast-feeding groups (0–6 months), 46–80% in 6–60 months of age and 37.8–97.5% in 5–20-year-old children.

**Comment 8**

*7 paragraph – 4rst sentence – in children, adults, or general population?*

*Response 8:  *We have now added ‘general population’ in the paragraph.

**Comment 9**

 8 paragraph – 4rst sentence – too long to comprehend. Please rewrite to improve clari4cation.

*Response 9:  *We thank the reviewer for his comment, we have paraphrased the sentence to a shorter and clearer one.

**Comment 10**

 8 paragraph – second sentence - What is the prevalence of vitamin D de4ciency among adults and children globally and in the UAE?

*Response 10:  *We have answered this comment from the reviewer in the 4 ^th^ paragraph of our introduction.

**Comment 11**

 9 paragraph – I would suggest write a broader aim and then the objectives that this study tried to achieve

*Response:  *We thank the reviewer for his suggestion here. We have now mentioned our aim, the need of this study to be conducted, and the high prevalence of vitamin D deficiency in the above paragraphs in a better pattern.

**Comment 12**

*Also, the phrase ‘among children in the general public’ sounds a bit odd to me. I think ‘among children’ in just one.*

*Response: *We agree with the reviewer and have omitted the phrase ‘in the general public’

**Comment 13**

*Methods*

*Can authors provide some examples of the public places that were used?*

*Response: *Yes, we have provided some examples of the public places that were used in our study.

**Comment 14**

*Can authors provide some references from where the questionnaire was adapted? Where were those studies conducted? Are there any cultural differences? How did the authors account for that?*

*Response: *Yes, we have provided the reference for the other study that we adapted the questionnaire from. We have also mentioned where the other study was conducted. However, our study has no association with the other study’s results or population. We have only adapted some of the questions from that study’s questionnaire.

**Comment 15**

*How many questions were there in total and in each section?*

*Response: *Thanking the reviewer for this keen observation. We have now mentioned the total number of questions in our questionnaire and the number of sections as well.

**Comment 16**

*Which section had yes/no and multiple response questions? Were there mixed question in each section?*

*Response: *Sections apart from the demographics, consisted of multiple responses and yes/no questions. Yes there were mixed sort of questions in some sections too.

**Comment 17**

*How did the authors evaluate the barriers to supplementation? The three sections do not tell anything about the barriers.*

*Response: *We thank the reviewer for his very thoughtful suggestion. It was important to mention about the barriers the parents face with providing VD supplementation to their children.

**Comment 18**

*How did the authors investigate the reasons for vitamin D de4ciency? I am not sure it was mentioned earlier until this point.*

*Response: *Thank you for this suggestion. We have rephrased the sentence and mentioned how we investigated the reasons for VDD.

**Comment 19**

*I am not sure what the authors are trying to tell through the primary outcomes. The 4rst two points look like the objectives. The third point looks like a hypothesis, but I am not sure what that means.*

*Response: *Thank you for this comment, we have paraphrased the primary outcomes in the manuscript.

**Comment 20**

*Around 319 participants were expected – Are the authors trying to imply that 319 participants were contacted?*

 Response: Thank you for this suggestion. We have rephrased the sentence to make better understanding for the reader on the number of study participants determined by Rao soft sample size calculator and the number of participants who responded and actively participated in our study.

**Comment 21**

 Response rate is excellent. How do the authors compare this with the similar other studies?

*Response: *Thank you for this comment, association between the response rate and the sample size is such that the response rate increases when the sample size less than 300 participants.

**Comment 22**

 Missing data – surprising!

*Response: *We have rephrased this to A total of 248 participants were included and all 248 of them actively participated in our study. During analysis, we found no missing data from our participants.

***Comment 23***

 • I suggest authors should mention earlier in the methods section that parents completed the survey.

*Response: *Thank you for suggesting, we have done this now.

**Comment 24**

 • Whether the questionnaire was self-administered or interviewer-administered

*Response: *Mentioned now in the manuscript that it was interviewer administered. Thank you.

**Comment 25**

 Looking at the information presented in ‘consent form’, it appears that the questionnaire was self-administered. Did the parents completed the questionnaire right there and then? How did they return the questionnaire?

*Response: *The questionnaire was both self-administered and interviewer-administered. The questionnaire content was described before letting the participants administer the data into it and the written consent form was taken from each participant prior to interviewing/ handing out the questionnaire.

**Comment 26**

 Why the authors excluded children with minor illnesses as well as those with cognitive impairment? It was parents who completed the survey

*Response: *The data was filled by the parents/ care givers but it was about children. Therefore, to avoid bias between the healthy children and ill children we excluded children with minor illnesses and those with cognitive impairment.

**Comment 27**

 Both a tabular and graphic version of the data was used to show it – generally we use one form of representation to avoid duplication

*Response: *Thank you for this suggestion but we have not used both the tabular and graphic version for one same piece of data. The frequency of nationality bar graph is different than that of tabular form, where the nationality is associated with supplementation practice.

**Comment 28**

*Bias – Given this is an observational study, how can the authors assure this*

 Response: Thank you for this suggestion. We have clarified our bias now.

***Comment 28***

*Authors did not mention about the language in which the questionnaire was designed and distributed to the participants*

 Response: Thank you, we have now mentioned that the article was generated in both English and Arabic

***Comment 29***

*Results 1 sentence – It was mentioned earlier. Avoid duplication.*

*Response: *Thank you. Duplication undone.

**Comment 30**

 2 sentence – I think the brackets are not utilised appropriately.

*Response: *Thank you for this comment, we have looked into it.

**Comment 31**

 From what I understand, 203 mothers and 39 fathers participated in the study. It makes a total of 242 participants. What about other 6 participants?

*Response: *Thank you for this correction. We have recalculated and put in the right figures.

**Comment 32**

 • If 203 is the correct number, it is 81.8% of the total participants not 89.1%.

*Response: *The calculation and figures are now corrected. We have put the right percentage now. Thank you.

**Comment 33**

 As per the information provided, 89.1% were mothers and 15.7% were fathers. It gives a total of 104.8%. Please check these numbers again

*Response: *Thank you. The correction for percentages of mothers and fathers in our study has now been corrected.

**Comment 34**

 • I strongly suggest authors to review their calculations and rewrite the results section.   

*Response: *Thank you for this suggestion, we appreciate your concern. We have reanalyzed our numbers and percentages and now have put in the right numbers. We have re-written our results.

**Comment 35**

 The name of countries in Fig 1 was not readable

*Response: *Thank you, we have added a readable picture.

**Comment 36**

 It was mentioned earlier that chi-square test was used but the results section did not mention chi-square values and any signi4cance values.

*Response: *the result part has been revised accrodingly

**Comment 37**

 The 4rst 3 paragraphs of the discussion are simply results. I suggest authors to summarise the key results in the 4rst paragraph to avoid duplication.

*Response: *Thank you for your highly appreciated suggestion. We have summarized the discussion part to a concise and clear paragraph now.

**Comment 38**

*• I think the discussion section needs considerable revision. Currently, it seems like a results section. I suggest authors to focus more explaining the results in light of the broader literature on the topic*

*Response: *Yes, we agree with the reviewer’s comment. Therefore, we have significantly made improvement in this particular section of our manuscript.

**Comment 39**

*The results of this study are generalizable – this statement needs more clari4cation. Earlier authors discussed the climate issues is UAE which is a contributing factor. Is the climate same in different parts of the world? Europe/US/Australia/Africa? Is not, how will the authors support their statement on generalisability?*

*Response: *Yes, we appreciate and agree with this comment. We have changed the phrase to- The results of this study are generalizable since it has been conducted on general population of children from multiple ethnicities in the U.A.E.

**Comment 40**

*Conclusion*

*I suggest authors to reconsider their conclusion. I am not sure what authors are….*

*Response: *Thank you so much for pointing out this suggestion to us. We have rephrased our conclusion to a very clear cut, understandable one now

# Peer review: Reviewer response for version 1

**DOI:** 10.5256/f1000research.135473.r157818

**Published:** 2022-12-14

**Authors:** Tauqeer Hussain Mallhi

**Affiliations:** 1 Department of Clinical Pharmacy, College of Pharmacy, Jouf University, Al-Jouf, Saudi Arabia

Thank you for the invitation to review this study. The authors made a good effort to explore the use of vitamin D supplementation among children in UAE. I have a few comments on this manuscript.

1. The sample size estimation can be omitted from the abstract section.
2. Please provide a few references that aid in the development of data collection form.
3. Please provide information on the validation and reliability of the data collection form.
4. Please clarify that the data was collected through interviews or self-administration techniques.
5. The quality of figure 1 can be improved.
6. Convenient sampling is also the limitation of this study that precludes the generatability of the findings.
7. There is a need to provide some information on the barriers of vitamin d supplementation in children. 
8. The manuscript will require some corrections in syntax at some places.

Is the work clearly and accurately presented and does it cite the current literature?

Yes

If applicable, is the statistical analysis and its interpretation appropriate?

Yes

Are all the source data underlying the results available to ensure full reproducibility?

Yes

Is the study design appropriate and is the work technically sound?

Yes

Are the conclusions drawn adequately supported by the results?

Yes

Are sufficient details of methods and analysis provided to allow replication by others?

Yes

Reviewer Expertise:

Pharmacy Practice, Clinical Pharmacy, Pharmacotherapy

I confirm that I have read this submission and believe that I have an appropriate level of expertise to confirm that it is of an acceptable scientific standard, however I have significant reservations, as outlined above.

# Response

**Published:** 2023-02-23

**Authors:** Syed Wasif Gillani

**Affiliations:** Gulf Medical University, United Arab Emirates

*Comment 1: The sample size estimation can be omitted from the abstract section.*

 Response 1: we agree with this comment therefore; we have accordingly omitted the sample size estimation from abstract of the manuscript.

*Comment 2: Please provide a few references that aid in the development of data collection form.*

 Response 2: Thank you for this suggestion. The references list is updated and the new references are incorporated in the list to emphasize this point.

*Comment 3: Please provide information on the validation and reliability of the data collection form.*

 Response 3: Thank you for pointing this out. We have added the suggested content to the manuscript.

*Comment 4: Please clarify that the data was collected through interviews or self-administration techniques.*

 Response 4: We agree with the reviewer’s assessment. Accordingly, throughout the manuscript, we have clarified that the data was collected on a questionnaire which was filled by the investigators. The investigator verbally inquired questions to the included parents individually from the questionnaire and filled the data on their behalves as they answered

*Comment 5: The quality of figure 1 can be improved.*

 Response 5: As suggested by the reviewer, we have updated figure 1 with improved quality in the results section of the manuscript.

*Comment 6: Convenient sampling is also the limitation of this study that precludes the*

*generatability of the findings.*

 Response 6: We agree that this is a potential limitation of the study. We have added this as a limitation in the discussion part of the manuscript.

*Comment 7: There is a need to provide some information on the barriers of vitamin d*

*supplementation in children.*

 Response 7: We agree with this and have incorporated your suggestion throughout the manuscript.
